# Patterns of medication use and factors associated with antibiotic use among adult fever patients at Singapore primary care clinics

**DOI:** 10.1186/s13756-016-0146-z

**Published:** 2016-11-24

**Authors:** Zaw Myo Tun, Mahesh Moorthy, Martin Linster, Yvonne C. F. Su, Richard James Coker, Eng Eong Ooi, Jenny Guek-Hong Low, Gavin J. D. Smith, Clarence C. Tam

**Affiliations:** 1 Saw Swee Hock School of Public Health, National University of Singapore, 12 Science Drive 2, #10-01, Tahir Foundation Building, Singapore, 117549 Singapore; 2 Duke-National University of Singapore Medical School, 8 College Road, Singapore, 169857 Singapore; 3 London School of Hygiene and Tropical Medicine, Keppel St, London, WC1E 7HT UK; 4 Department of Infectious Disease, Singapore General Hospital, Outram Road, Singapore, 169608 Singapore

**Keywords:** Antimicrobial resistance, Antibiotic use, Acute febrile illness, Primary care, Singapore

## Abstract

**Background:**

Antimicrobial resistance is a public health problem of global importance. In Singapore, much focus has been given to antibiotic usage patterns in hospital settings. Data on antibiotic use in primary care is lacking. We describe antibiotic usage patterns and assess factors contributing to antibiotic usage among adults presenting with acute febrile illness (AFI) in primary care settings in Singapore.

**Methods:**

We analyzed data from the Early Dengue infection and outcome study. Adults with AFI presenting at 5 Singapore polyclinics were included. We used multivariable logistic regression to assess demographic, clinical and laboratory factors associated with antibiotic usage among adults with AFI.

**Results:**

Between December 2007 and February 2013, 1884 adult AFI patients were enrolled. Overall, 16% of adult AFI patients reported antibiotic use. We observed a rise in the use of over-the-counter medications in late 2009 and a decrease in antibiotic use during 2010, possibly related to the outbreak of pandemic influenza A H1N1 virus. After adjusting for age, gender, polyclinic and year of enrolment, the following factors were associated with higher odds of antibiotic use: living in landed property (compared to public housing) (OR = 1.73; 95% CI: 1.06–2.80); body mass index (BMI) <18.5 (OR = 1.87; 95% CI: 1.19–2.93); elevated white blood cell (WBC) count (OR = 1.98; 95% CI: 1.42–2.78); and persistence of initial symptoms at 2–3 days follow-up with OR (95% CI) for categories of 1, 2, 3, and ≥4 persisting symptoms being 2.00 (1.38–2.92), 2.67 (1.80–3.97), 4.26 (2.73–6.64), and 2.79 (1.84–4.24) respectively.

**Conclusions:**

Our study provides insights on antibiotic usage among adult patients presenting to primary care clinics with febrile illness, and suggests that high socio-economic status, and risk factors of a severe illness, that is, low BMI and persistence of initial symptoms, are associated with higher antibiotic use. Further work to understand trends of antibiotic usage in both private and public primary care clinics, and factors that influence patient expectation and physician prescribing of antibiotics is important.

## Background

Antimicrobial resistance (AMR) is a public health problem of global importance. The recent O’Neill review on AMR, commissioned by the government of the United Kingdom, suggests that the death toll from antimicrobial-resistant infections could exceed 10 million annually by 2050, surpassing mortality from cancer [1]. Antibiotic use is associated with AMR. [2] A systematic review by Costelloe and colleagues showed that the greater the number or duration of antibiotics prescribed in previous 12 months, the greater the likelihood of isolating drug-resistant bacteria [3]. Thus, it is important to optimize the use of antibiotics in humans and animals. This was also a particular focus of the O’Neill review and a key recommendation in the 2015 World Health Organization (WHO) Global Action Plan on AMR. [1, 4] Understanding levels and determinants of antibiotic use is critical to inform guidelines on optimal antibiotic use. Previous studies from the United States and the Netherlands reported excessive and inappropriate use of antibiotics in primary care [5, 6]. In Hong Kong, level of antibiotic use in primary care was generally similar to other developed settings with relatively lower number of antibiotics used among patients with upper respiratory tract infections [7]. In Singapore, much focus has been given to antibiotic usage patterns in hospital settings while data on antibiotic usage in primary care are lacking [8, 9]. In this paper, we report antibiotic usage patterns and factors associated with antibiotic usage among adult fever patients presenting to primary care clinics in Singapore.

## Methods

### Study population

We analyzed demographic, clinical and epidemiological data from adults with acute febrile illness (AFI) enrolled in the Early DENgue infection and outcome (EDEN) study [10]. The aim of the EDEN study was to characterize epidemiological, clinical, viral, and host specific features of adult dengue disease. It was conducted at five Singapore polyclinics which are government-subsidized, comprehensive primary care clinics. Polyclinics cater for approximately a quarter of primary care attendance in Singapore [11]. Adults (age ≥17 years) presenting to the study polyclinics between December 2007 and February 2013 with a history of fever (body temperature ≥38 °C) for less than 72 h were enrolled. Data were collected using standardized data forms. Participants were followed up on 2 separate occasions at 2–3 days and 4 weeks from the initial visit. Blood samples were collected at each visit for a range of laboratory diagnostics. In this analysis, AFI was defined as fever (body temperature ≥38 °C) for not longer than 72 h at initial presentation. Influenza-like illness (ILI) was defined as fever and cough according to the WHO definition [12]. Hospital admissions that took place within 5 days of the initial visit were considered due to the current AFI episode. Antibiotic use was defined as patients’ self-reported use of antibiotics for the illness.

### Statistical analysis

We described the frequency of antibiotic usage among AFI patients, and examined the use of over-the-counter (OTC) medications (i.e. analgesics, cough medications and cold remedies) and antibiotics over time. Discrete and continuous variables were summarized using mean (standard deviation, SD) or median and (interquartile range, IQR) as appropriate; categorical variables were summarized using frequencies and percentages. We investigated associations between antibiotic use among AFI patients and demographic characteristics, clinical symptoms and laboratory parameters using *χ* ^2^ statistics and *p*-values.

We used multivariable logistic regression to identify factors independently associated with antibiotic use. In a sub-group analysis, we further examined these factors among patients meeting the WHO definition for ILI. Variables with *p*-value <0.2 in the univariable analysis were considered for inclusion in multivariable analysis. We included age group, gender, recruiting polyclinic, and year of enrolment as covariates in all models. We added explanatory variables in a forward step-wise procedure, using the likelihood ratio (LR) test to assess the contribution of each variable to the model. We retained variables with a LR test *p*-value <0.05 and summarized the association between each variable and antibiotic usage with odds ratios (ORs) and 95% confidence intervals (CIs). Statistical analysis was performed using Stata 12 (Stata Corp).

## Results

We analyzed data from 1884 adult AFI patients enrolled in the EDEN study between December 2007 and February 2013. The mean age of patients was 36.7 years (SD, 14.7), 1235 (66%) were male, and 958 (51%) were Chinese. Most patients (74.3%) lived in public housing. Average body temperature at initial presentation was 38.2 °C, median duration of illness was 5 days (IQR, 3–7), and 759 (40%) patients satisfied the WHO definition of ILI. Less than 5% of AFI patients were known to have diabetes; other comorbidities (i.e. malignancy, and ischemic heart disease) were uncommon (<1%) (Table 1).

**Table 1:** Characteristics and Antibiotic Use of 1884 Fever Patients Presenting at Singapore Polyclinics, December 2007-February 2013

Characteristics	N (%)		Antibiotic use, n (%)		*p-value*
Age in years					
17–24	482	(25.6)	80	(16.6)	0.052
25–34	529	(28.1)	65	(12.3)	
35–44	309	(16.4)	55	(17.8)	
45–54	279	(14.8)	52	(18.6)	
55–64	193	(10.2)	37	(19.2)	
65 and above	92	(4.9)	20	(21.7)	
Gender					
Female	649	(34.4)	129	(19.9)	0.003
Male	1235	(65.6)	180	(14.6)	
Year of enrolment					
Dec 2007–2008	146	(7.7)	28	(19.2)	0.003
2009	232	(12.3)	53	(22.8)	
2010	611	(32.4)	76	(12.4)	
2011	576	(30.6)	102	(17.7)	
2012-Feb 2013	319	(16.9)	50	(15.7)	
Recruiting polyclinic					
Polyclinic A	928	(49.3)	220	(23.7)	<0.001
Polyclinic B	620	(32.9)	52	(8.4)	
Polyclinic C	193	(10.2)	19	(9.8)	
Polyclinic D	101	(5.4)	5	(5.0)	
Polyclinic E	42	(2.2)	13	(31.0)	
Body Mass Index (kg/m^2^)					
<18.5	162	(8.6)	39	(24.1)	<0.001
18.5–22.9	798	(42.4)	111	(13.9)	
23–27.4	634	(33.7)	93	(14.7)	
≥27.5	290	(15.4)	66	(22.8)	
Ethnicity					
Chinese	958	(50.8)	182	(19.0)	0.001
Indian	312	(16.6)	39	(12.5)	
Malay	330	(17.5)	59	(17.9)	
Other	283	(15.0)	29	(10.2)	
Missing values	1	(0.1)			
Housing type					
Condominium	66	(3.5)	13	(19.7)	<0.001
Dormitory/Hostel	228	(12.1)	9	(3.9)	
Public housing (HDB flat)	1399	(74.3)	251	(17.9)	
Landed Property	118	(6.3)	29	(24.6)	
Work site	71	(3.8)	7	(9.9)	
Missing values	2	(0.1)			
Duration of illness					
≤5 days	1053	(55.9)	135	(12.8)	<0.001
>5 days	760	(40.3)	168	(22.1)	
Missing values	71	(3.8)			
Diabetes					
No	1797	(95.4)	286	(15.9)	0.01
Yes	87	(4.6)	23	(26.4)	
Initial body temperature (° C)					
<38	596	(31.6)	83	(13.9)	0.047
38–38.9	921	(48.9)	153	(16.6)	
≥39	365	(19.4)	73	(20.0)	
Missing values	2	(0.1)			
Number of initial symptoms other than fever					
0–2	280	(14.9)	27	(9.6)	0.002
3–4	487	(25.8)	74	(15.2)	
5–7	657	(34.9)	118	(18.0)	
8–15	460	(24.4)	90	(19.6)	
Number of initial visit symptoms persisting at the first follow-up^a^					
0	821	(43.6)	79	(9.6)	<0.001
1	358	(19.0)	64	(17.9)	
2	264	(14.0)	60	(22.7)	
3	154	(8.2)	52	(33.8)	
≥4	246	(13.1)	54	(22.0)	
Missing values	41	(2.2)			
Occurrence of new symptoms reported at the first follow-up^a^					
0	1214	(64.4)	175	(14.4)	0.001
1	372	(19.7)	76	(20.4)	
2	155	(8.2)	32	(20.6)	
≥3	102	(5.4)	26	(25.5)	
Missing values	41	(2.2)			
Influenza-like illness^b^					
No	1123	(59.6)	160	(14.2)	0.002
Yes	759	(40.3)	149	(19.6)	
Missing values	2	(0.1)			
Type of employment					
Not employed	365	(19.4)	61	(16.7)	0.284
Blue collar	1032	(54.8)	155	(15.0)	
White collar	438	(23.2)	83	(18.9)	
Other	47	(2.5)	9	(19.1)	
Missing values	2	(0.1)			
Season					
NEM	479	(25.4)	77	(16.1)	0.821
PSWM	395	(21.0)	63	(15.9)	
SWM	709	(37.6)	114	(16.1)	
PNEM	301	(16.0)	55	(18.3)	
Hospitalization as a result of febrile illness					
Not hospitalized	1831	(97.2)	301	(16.4)	0.354
Hospitalized	53	(2.8)	8	(15.1)	
WBC count at initial visit (×10^9^ cells/L)					
Leukopenia *(<4)*	204	(10.8)	26	(12.7)	0.001
Normal range *(4–11)*	1362	(72.3)	210	(15.4)	
Leukocytosis *(>11)*	305	(16.2)	71	(23.3)	
Missing values	13	(0.7)			
Anemia at initial visit (hemoglobin concentration in g/dl)^#^					
No anemia *(male, ≥13; female, ≥12)*	1685	(89.4)	281	(16.7)	0.702
Mild anemia *(male, 11–12.9; female, 11–11.9)*	121	(6.4)	17	(14.0)	
Moderate anemia *(8–10.9)*	48	(2.5)	6	(12.5)	
Severe anemia *(<8)*	10	(0.5)	1	(10.0)	
Missing values	20	(1.1)			
Platelet count at initial visit (plt/μL)					
Thrombocytopenia *(<150)*	279	(14.8)	42	(15.1)	0.609
Normal range *(150–400)*	1547	(82.1)	260	(16.8)	
Thrombocytosis *(>400)*	40	(2.1)	5	(12.5)	
Missing values	18	(1.0)			

*HDB* Housing and Development Board, *NEM* Northeast Monsoon Season (December to mid-March), *PSWM* Pre Southwest Monsoon (mid-March to May), *SWM* Southwest Monsoon (June to September), *PNEM* Pre Northeast Monsoon (October to November), *WBC* White Blood Cells

^a^2–3 days after the initial visit

^b^Influenza-like illness is defined as fever of ≥38 °C and cough according to WHO [12]

^#^Anemia as defined by haemoglobin concentration cut-offs from World Health Organization [28]

**Fig. 1:**
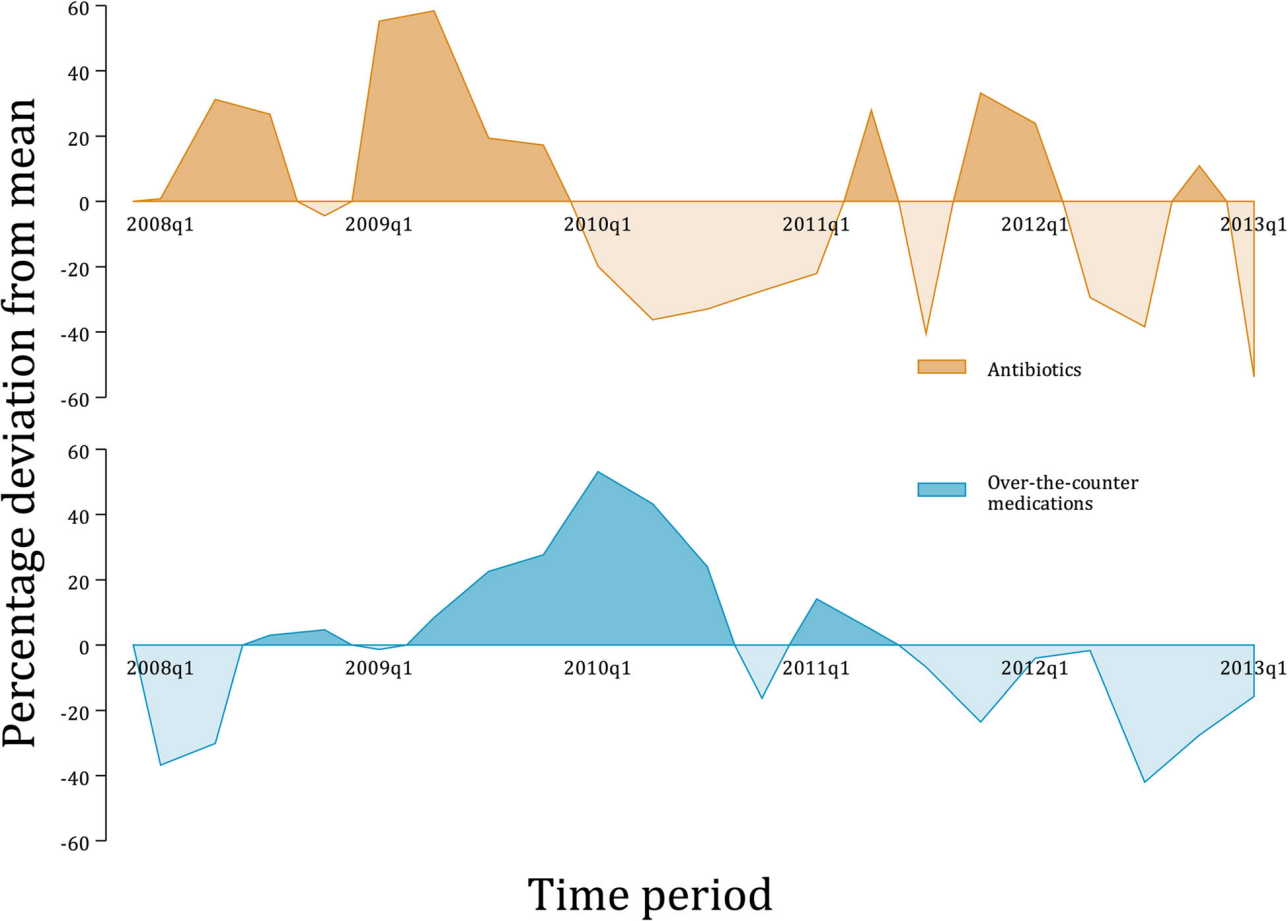
Use of Antibiotics and Over-the-Counter Medications Between December 2007 and February 2013

Overall, 309 (16%) patients used antibiotics, ranging from 5 to 31% across 5 polyclinics. OTC medication was used by 56% of patients, and included use of analgesics (309, 16%), cough medicines (608, 32%) and cold remedies (378, 20%). Figure 1 presents the use of antibiotics and over-the-counter medication over time. A marked drop in the percentage of patients who used antibiotics occurred in 2010, with a reduction of 40% from the mean for the entire study period in the second quarter of 2010. This coincided with increased use of OTC medication. This effect was only temporary and was reversed from 2011 onwards.

**Fig. 2:**
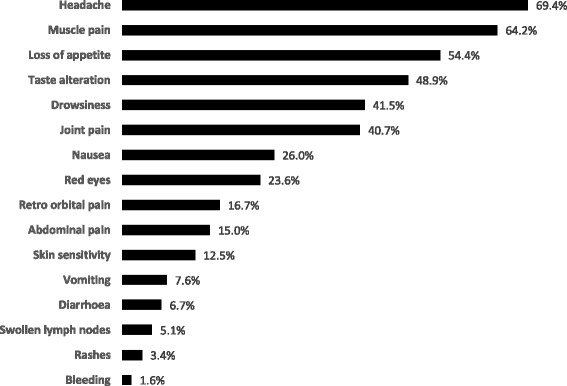
Symptom profile of 1884 Fever Patients Presenting at Singapore Polyclinics, December 2007-February 2013

Among AFI patients, 94% reported two or more symptoms at the initial presentation. Headache (69%), muscle pain (64%), and loss of appetite (54%) were the three most commonly reported symptoms. Vomiting, diarrhea, swollen lymph nodes, rashes, and bleeding were each reported by fewer than 10% of patients (Fig. 2).

In multivariable analysis, factors associated with increased odds of antibiotic use, after adjusting for age, gender, recruiting polyclinic, and year of enrolment, included living in landed property compared to public housing (OR = 1.73; 95% CI: 1.06–2.80); having a body mass index (BMI) below 18.5 kg/m^2^ (OR = 1.87; 95% CI: 1.19–2.93), and elevated WBC count (OR = 1.98; 95% CI: 1.42–2.78). Persistence of symptoms at 2–3 days from initial presentation was also associated with increased odds of antibiotic use; adjusted OR (95% CI) for categories of 1, 2, 3, and ≥4 persisting symptoms were 2.00 (1.38–2.92), 2.67 (1.80–3.97), 4.26 (2.73–6.64), and 2.79 (1.84–4.24) respectively (Table 2).

**Table 2:** Factors Associated with Antibiotic Use among 1884 Fever Patients at Febrile Polyclinics, December 2007-February 2013

Characteristics	Unadjusted OR (95% CI)	*p-value*	Adjusted OR (95% CI)	*p-value*
Age (years)^a^				
17–24	1	0.0463	1	0.7211
25–34	0.70 (0.49–1.00)		0.95 (0.64–1.41)	
35–44	1.09 (0.75–1.59)		1.26 (0.83–1.93)	
45–54	1.15 (0.78–1.69)		1.14 (0.74–1.76)	
55–64	1.19 (0.77–1.83)		1.14 (0.70–1.84)	
65 and above	1.40 (0.80–2.42)		0.83 (0.45–1.55)	
Gender^a^				
Female	1	0.0035	1	0.5817
Male	0.69 (0.54–0.88)		0.92 (0.69–1.23)	
Year of enrolment^a^				
Dec 2007–2008	1	0.0037	1	0.0177
2009	1.25 (0.75–2.09)		1.83 (1.04–3.23)	
2010	0.60 (0.37–0.96)		1.37 (0.79–2.36)	
2011	0.91 (0.57–1.44)		2.03 (1.18–3.47)	
2012-Feb 2013	0.78 (0.47–1.31)		1.24 (0.70–2.21)	
Polyclinic enrolled^a^				
Polyclinic A	1	<0.0001	1	<0.0001
Polyclinic B	0.17 (0.07–0.42)		0.22 (0.09–0.57)	
Polyclinic C	0.29 (0.21–0.41)		0.32 (0.22–0.45)	
Polyclinic D	0.35 (0.21–0.58)		0.32 (0.18–0.54)	
Polyclinic E	1.44 (0.74–2.82)		1.74 (0.80–3.78)	
Body Mass Index (kg/m^2^)				
<18.5	1.98 (1.31–2.99)		1.87 (1.19–2.93)	
18.5–22.9	1	0.0117	1	0. 0106
23–27.4	1.08 (0.81–1.46)		0.91 (0.66–1.27)	
≥27.5	1.86 (1.32–2.60)		1.31 (0.90–1.92)	
Housing type				
Public housing (HDB flat)	1	0.0270	1	0.0045
Condominium	1.08 (0.58–2.00)		1.02 (0.51–2.03)	
Dormitory/Hostel	0.19 (0.09–0.37)		0.38 (0.18–0.79)	
Landed property	1.47 (0.94–2.28)		1.73 (1.06–2.80)	
Work site	0.49 (0.22–1.08)		0.70 (0.30–1.64)	
Number of initial symptoms persisting at first follow-up				
0	1	0.0414	1	<0.0001
1	2.00 (1.40–2.85)		2.00 (1.38–2.92)	
2	2.72 (1.88–3.92)		2.67 (1.80–3.97)	
3	4.66 (3.11–6.98)		4.26 (2.73–6.64)	
4 and above	2.57 (1.76–3.75)		2.79 (1.84–4.24)	
WBC count (cells/μL) at initial visit				
Leukopenia *(<4)*	0.83 (0.51–1.35)		0.61 (0.38–0.99)	
Normal range *(4–11)*	1	0.0097	1	<0.0001
Leukocytosis *(>11)*	1.73 (1.19–2.51)		1.98 (1.42–2.78)	
Headache at initial visit				
No	1	0.0021	1	0.0239
Yes	0.78 (0.60–1.01)		0.73 (0.54–0.97)	

*HDB* Housing and Development Board, *OR* odds ratio, *WBC* white blood cell

^a^Variables included *a priori* as covariates in the model

In a sub-group analysis of patients who met the WHO definition of ILI, after adjusting for age, gender, recruiting polyclinic, and year of enrolment, an elevated WBC count at initial presentation (OR = 1.97; 95% CI: 1.17–3.33), and persistence of symptoms at 2–3 days from initial presentation [(OR = 1.42; 95% CI: 0.79–2.57), (OR = 2.30; 95% CI: 1.23–4.30), (OR = 5.12; 95% CI: 2.67–9.83), and (OR = 2.28; 95% CI: 1.24–4.19) for categories of 1, 2, 3 and ≥4 persisting symptoms respectively] were associated with higher odds of antibiotic usage (Table 3).

**Table 3:** Subgroup Analysis of Patients Who Met the Influenza-like Illness definition [12] at Polyclinics, December 2007-February 2013

Characteristics	Unadjusted OR (95% CI)	*p value*	Adjusted OR (95% CI)	*p value*
Age (years)^a^				
17–24	1	0.6777	1	0.9438
25–34	0.77 (0.46–1.28)		0.84 (0.48–1.49)	
35–44	0.96 (0.55–1.70)		0.94 (0.50–1.77)	
45–54	0.89 (0.50–1.60)		0.79 (0.41–1.51)	
55–64	1.24 (0.69–2.25)		1.11 (0.56–2.17)	
65 and above	1.25 (0.55–2.87)		0.92 (0.37–2.34)	
Gender^a^				
Female	1	0.0147	1	0.1394
Male	0.64 (0.44–0.91)		0.72 (0.47–1.11)	
Year of enrolment^a^				
Dec 2007–2008	1	0.0021	1	0.0239
2009	0.91 (0.29–2.84)		1.45 (0.42–4.94)	
2010	0.37 (0.13–1.12)		0.62 (0.19–1.97)	
2011	0.90 (0.31–2.64)		1.20 (0.39–3.71)	
2012-Feb 2013	0.56 (0.19–1.68)		0.68 (0.22–2.15)	
Polyclinic enrolled^a^				
Polyclinic A	1	<0.0001	1	<0.0001
Polyclinic B	0.22 (0.14–0.37)		0.24 (0.14–0.41)	
Polyclinic C	0.39 (0.2–0.77)		0.31 (0.14–0.67)	
Polyclinic D and E^b^	0.76 (0.21–2.82)		0.61 (0.15–2.46)	
Number of initial visit symptom persisting at first follow-up				
0	1	<0.0001	1	<0.0001
1	1.68 (0.96–2.93)		1.42 (0.79–2.57)	
2	2.48 (1.38–4.45)		2.30 (1.23–4.30)	
3	6.55 (3.59–11.94)		5.12 (2.67–9.83)	
4 and above	2.37 (1.34–4.17)		2.28 (1.24–4.19)	
WBC count at initial visit (cells/μL)				
Leukopenia *(<4)*	1.07 (0.57–2.04)		0.99 (0.49–2.01)	
Normal range *(4–11)*	1	0.1440	1	0.0433
Leukocytosis *(>11)*	1.61 (1.01–2.55)		1.97 (1.17–3.33)	

*OR* odds ratio

^a^Variables included *a priori* as covariates in the model

^b^Polyclinic D and E were combined because of low number of patients (5 and 8 respectively)

## Discussion

We conducted a secondary data analysis, and assessed antibiotic use among adult fever patients in primary care setting, and factors associated with elevated antibiotic use. We found that overall 16% of adult AFI patients reported antibiotic use, although there was considerable variation between polyclinics. Factors that are likely to indicate higher socioeconomic status, and factors that are risk factors of a severe illness were associated with higher odds of antibiotic use.

Our estimates of antibiotic usage are difficult to compare to other settings, as previous studies have reported antibiotic usage or prescription overall, rather than specifically in adult fever patients. A Malaysian study by Ab Rahman and colleagues showed that 24% of adults were prescribed antibiotic in primary care clinics [13]. A survey conducted in Indonesia reported that an overall 21% patients attending public primary care centers consumed antibiotic [14]. In the United States, antibiotic prescription was reported in 10% of overall ambulatory visits [15].

Our study shows that patients living in a landed property were more likely to use antibiotics. In Singapore, landed properties (such as terrace houses, semi-detached houses, and bungalows) are likely to be related to higher socioeconomic status [16]. A possible explanation to our finding is that patients of higher socioeconomic status were more likely to expect an antibiotic prescription from physicians. This is consistent with the findings of a study from the United States [17].

Having low BMI and persisting symptoms at 2–3 days from the initial presentation were associated with a higher odds of antibiotic usage. Prescribing clinicians may perceive these as risk factors for more severe or longer-lasting illness, or secondary infection [18–20], and may be more likely to prescribe antibiotics to these patients [6, 21].

Fever patients with elevated WBC count at initial presentation were more likely to use antibiotics. A similar association was also observed in the subgroup of patients with ILI. A high WBC count might indicate a bacterial infection, and thus the likelihood of antibiotic prescription. Studies from Switzerland and China found that more antibiotic use among fever patients was assoiated with higher WBC count [22, 23].

Our data indicate that antibiotic use decreased markedly in late 2009 and 2010, during which time the use of OTC medications increased. This coincided with the period following the epidemic of influenza A H1N1, which was introduced into Singapore in May 2009 and peaked in the latter part of 2009 and early 2010 [24]. The reduction in antibiotic use during this period could be due to heightened awareness among primary care physicians of the H1N1 pandemic. A similar phenomenon was reported in a study by Hebert and colleagues, in which lower antibiotic prescribing was observed during a pandemic period of febrile respiratory illness in the metropolitan Chicago area. [25] Although this pattern of reduced antibiotic usage was reversed in 2011, it suggests that considerable reductions in antibiotic prescription are possible in primary care.

The EDEN study was primarily designed to assess Dengue virus infection patterns, and antibiotic use was self-reported by the participants. Data on prescription, type, dosage and timing of antibiotic use were not available. Certain respiratory symptoms, such as cough, were not systematically recorded, and may have led to an underestimation of ILI.

Among fever patients in Singapore, approximately 80% of primary care consultations take place at private institutions [11]. The EDEN study did not sample patients visiting private practitioners. Patients accessing public primary care services are likely to differ from those using private services. For example, there were notable differences between patients in our data and the general population: 50% of patients were Chinese and 72% lived in HDB flats compared to 74% and 82% of the general population, respectively [26, 27]. In addition, antibiotic prescription and usage patterns among patients using primary care services in the private sector may be different to those in the public sector. A Malaysian study reported higher antibiotic prescription among healthcare providers in the private sector compared to those in the public sector [13]. Inclusion of private healthcare providers in research studies is important to gain a comprehensive picture of antibiotic usage in primary care.

## Conclusions

Our study provides insights on antibiotic usage among adult patients presenting to primary care clinics with febrile illness, and suggests that high socio-economic status, and risk factors of a severe illness, that is, low BMI and persistence of initial symptoms, are associated with higher antibiotic use. Further work to understand trends of antibiotic use, both in the public and private sectors, as well as factors that influence patient expectation and physician prescribing of antibiotics is important, in order to identify opportunities to rationalize antibiotic use and develop policies for optimal antibiotic use in primary care.

## Abbreviations

AFI: Acute febrile illness
BMI: Body mass index
CI: Confidence intervals
EDEN: Early DENgue infection and outcome study
HDB: Housing and Development Board
ILI: Influenza-like illness
LR: Likelihood ratio
OR: Odds ratio
OTC: Over-the-counter
WBC: White Blood Cells
